# Mechanical stability of individual bacterial cells under different osmotic pressure conditions: a nanoindentation study of *Pseudomonas aeruginosa*

**DOI:** 10.3762/bjnano.16.86

**Published:** 2025-07-21

**Authors:** Lizeth García-Torres, Idania De Alba Montero, Eleazar Samuel Kolosovas-Machuca, Facundo Ruiz, Sumati Bhatia, Jose Luis Cuellar Camacho, Jaime Ruiz-García

**Affiliations:** 1 Coordinación para la Innovación y Aplicación de la Ciencia y la Tecnología, Universidad Autónoma de San Luis Potosí, 550 Sierra Leona Ave., 78210 San Luis potosí, SLP, México; 2 Facultad de Ciencias, Universidad Autónoma de San Luis Potosí, Parque Chapultepec 1570, Privadas del Pedregal 78295, San Luis Potosí, S.L.P. México; 3 Department of Chemistry, Faculty of Science and Engineering, Swansea University, Singleton Campus, Swansea SA2 8PP, United Kingdom; 4 Secretaría de Ciencia, Humanidades, Tecnología e Innovación, México; 5 Master program in Sciences and Material Engineering (MCIM-UAZ), Autonomous University of Zacatecas, 801 López Velarde St, 9800 Zacatecas, Mexico; 6 Instituto de Física, Parque Chapultepec 1570, Privadas del Pedregal 78295, San Luis Potosí, S.L.P. México

**Keywords:** AFM, force spectroscopy, membrane rigidity, nanomechanical mapping, osmotic shock

## Abstract

Nanomechanical maps to test the mechanical response of the outer envelope of *Pseudomonas aeruginosa* were obtained utilizing atomic force microscopy in force–volume mode in the low range of loading forces when exposed to hypotonic (Milli-Q water), isotonic (PBS), and hypertonic (0.5 M NaCl) solutions. Imaging and mechanical testing showed that bacteria are highly resilient to deformation and can withstand repetitive indentations in the range of 500 pN. Analysis of force spectra revealed that although there are differences in the mechanical response within the first stages of nanoindentation, similar values in the slopes of the curves reflected a stable stiffness of about *k*_B_ = 20 mN/m and turgor pressures of *P*_t_ = 12.1 kPa. Interestingly, a change in the nonlinear regime of the force curves and a gradual increase in maximal deformation by the AFM tip from hypotonic to hypertonic solutions suggest a softening of the outer envelope, which we associate with intense dehydration and membrane separation between inner and outer envelopes. Application of a contact mechanics model to account for the minute differences in mechanical behavior upon deformation provided Young’s moduli in the range of 0.7–1.1 kPa. Implications of the presented results with previously reported data in the literature are discussed.

## Introduction

*Pseudomonas aeruginosa* (PA) is a Gram-negative bacterium belonging to the *Pseudomonas* genus. It is well known for its versatility and adaptability in various environments, as it can be found in multiple habitats, including soil, water, plants, and animals. It can also prosper in artificial environments such as metal or plastic pipes and medical devices [1–5]. Although PA is part of the normal microbiota of the skin and mucous membranes of many healthy individuals, it can cause serious opportunistic infections in the respiratory and urinary tracts and during wound healing in people with weakened immune systems or in hospitalized patients [6–8]. As a Gram-negative bacterium, PA is characterized by a distinctive cell wall structure constituted by a thin peptidoglycan layer enclosed by an outer membrane which contains lipopolysaccharides (LPS) [9–11]. The outer membrane also contains numerous proteins, lipoproteins, and channels contributing to its selective permeability [12–13]. Also integrated into the outer membrane, specific tension-activated channel proteins are responsible for the osmoregulation of the membrane envelope and its protection from threatening conditions such as severe osmotic downshocks, which can lead to an excessive increase in the membrane tension resulting in rupture [14–15]. The mechanosensitive (MS) family of channel proteins have been identified as the main efflux pumps required by PA to regulate the exit of osmolytes and reduce the membrane tension to acceptable life-compatible values. Therefore, the type and density of these MS channels triggered at different threshold values of membrane tension determine the survival capacity of the bacteria under drastic changes in osmotic pressure [16–20]. PA is also known to use other channel proteins to overcome the attack by antibiotics via rapid extrusion of the uptaken drugs, which confers remarkable resistance to this pathogen [21–34]. To hinder this difficulty, natural and synthetic molecular inhibitors with high binding affinity towards these channel proteins have been proposed [25–32]. Likewise, the application of silver and copper nanoparticles to block these molecular pores has been reported for PA and other bacteria as well [33–37]. In the latter cases, a direct consequence of this highly controlled membrane permeability is that membrane tension and rigidity are two intertwined physical parameters with a dynamic behavior dictated by the internal turgor pressure of the bacteria during swelling or plasmolysis. Therefore, understanding the dynamics of their mechanical response due to changes in external conditions or exposure to specific molecular agents is critical in generating strategies to control their undesired propagation.

**Figure 1:**
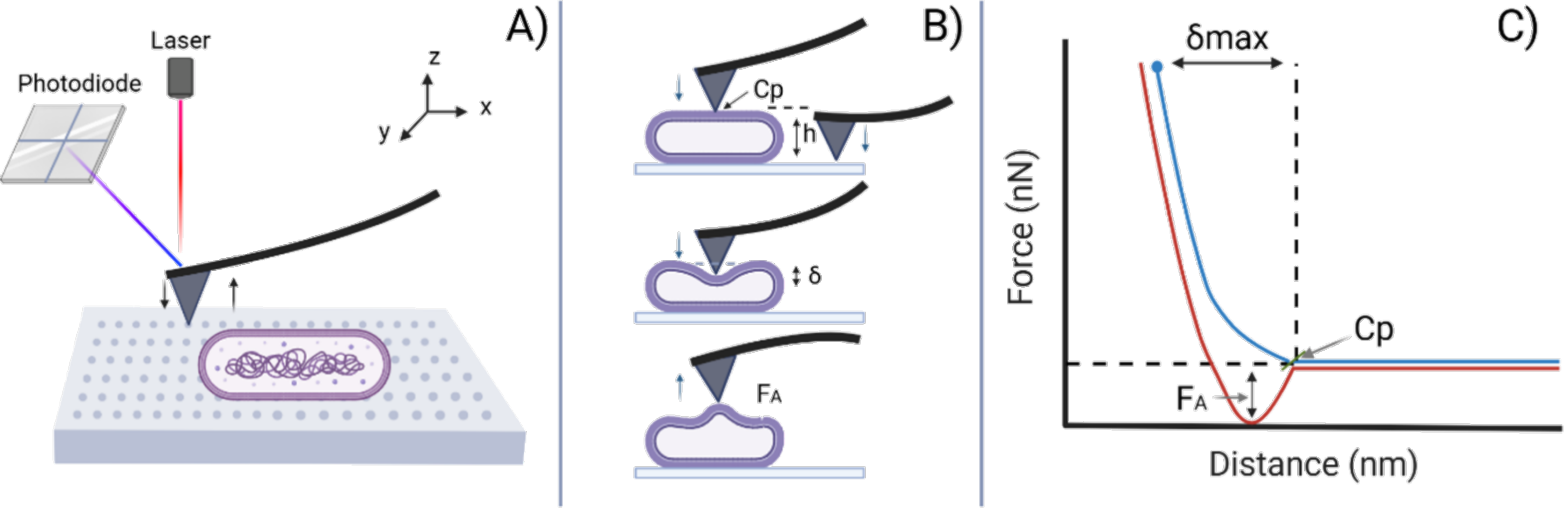
Schematic representation of the force–volume approach to quantify nanomechanical interactions with the atomic force microscope. A) A pre-selected grid that indicates the multiple specific points the AFM tip follows to perform individual indentations with a predefined maximal loading force. B) Drawing that shows how the tip indents on the sample surface in approach/retraction cycles, from which quantitative information such as height, stiffness, Young’s modulus, and adhesion are extracted. In C), a schematic illustration of a representative force–separation curve taken on a deformable sample shows the profile of the interaction of the AFM tip with the sample surface and its contact point, maximal deformation, and adhesion force. Created in BioRender. García-torres, L. (2025) https://BioRender.com/zpchrmj. This content is not subject to CC BY 4.0.

Atomic force microscopy (AFM) is a powerful, sensitive technique that scans the surface topography of a sample with an ultra-sharp tip while monitoring the interaction forces between this tip and the sample at the nanoscale. The force applied by the AFM tip on the sample is controlled by monitoring the deflection of an extremity of a micrometer mechanical lever onto which the tip is attached. In the study of pathogens, AFM excels in providing high-resolution topographic images while measurements are performed in solution in a fluid chamber under controlled environmental conditions. Thus, critical structural changes on the lifestyle of the pathogen can be investigated [38–42]. Beyond imaging, AFM force spectroscopy capabilities are essential to extract material properties of the investigated sample [11,43–44]. The force–volume (FV) mode allows for mapping surface physical parameters of the analyzed sample via controlled nanoindentations on a pre-programmed grid on the sample surface [44–47]. In conventional scanning modes (e.g., contact and tapping) the changes in displacement by the piezoelectric element are gathered in order to maintain a constant cantilever deflection or amplitude while a surface is scanned line by line Conversely, in FV quantitative information is extracted after analysis of a force–separation curve obtained from a performed nanoindentation. Therefore, the pre-programed grid in FV defines the amount of information (number of nanoindentations per scanned line) taken from the sample surface and also its resolution. Typical parameters obtained using the FV mode are the height, stiffness, adhesion, elasticity modulus, and dissipation of the sample. The mentioned parameters derived from force–separation curves are analyzed and mapped in real time as shown below in Figure 1. In the present study, we investigated the mechanical response of the outer membrane of PA at the single bacterium level in FV mode when exposed to relevant osmolarity conditions. Of particular interest was the extraction and comparison of nanomechanical maps obtained in the low range of loading forces to quantify its morphology, membrane stiffness, Young’s modulus of elasticity and adhesion when PA was tested in hypotonic (Milli-Q water), isotonic (0.1 M phosphate buffered solution), and hypertonic (0.5 M NaCl) solutions.

## Materials and Methods

### Bacterial culture

The strain of *Pseudomonas aeruginosa* (ATCC^®^ 27853™) was reactivated by transferring it to Mueller–Hinton broth and incubating it for 24 h at a temperature of 37 °C. Subsequently, it was cultured on Mueller–Hinton agar and again incubated for 24 h under the same conditions. Isolated colonies of PA were collected using a calibrated loop and suspended in 5 mL of phosphate buffer supplemented with 50 µL of Mueller–Hinton broth. The suspension was vortexed for 10 s before its preparation for AFM analysis.

### Sample preparation for atomic force microscopy

For AFM, the sample was initially firmly adhered to a substrate for subsequent scanning, with the tip first used to identify the objects of study on the surface. In this case, PA in suspension was adhered to a solid mica substrate, previously cleaved with regular adhesive tape, and coated with 5 μL of the cationic polymer poly-ʟ-lysine (PLL, MW 40 kDa, Sigma-Aldrich). The drop of PLL was not allowed to dry but incubated for 10 min, and then the surfaces were repeatedly rinsed with Milli-Q water to allow the formation of a thin film. This coating promotes bacterial adhesion through short-range electrostatic and hydrophobic interactions. Before deposition on the PLL-coated substrate, the bacterial suspension was centrifuged at 2500 rpm for 3 min, and the resulting supernatant was removed. The bacteria were then resuspended in 150 μL of PBS to increase their concentration and before the previous deposition of 5 μL on the PLL-coated substrate. A liquid cell was assembled to measure changes in bacterial membrane rigidity response when transitioning between different solutions. Once inside the fluid cell chamber, bacteria were imaged and tested in PBS solution and then exposed to the desired osmolarity conditions for investigation. The applied solutions were ultrapure water (Milli-Q), phosphate-buffered saline solution (PBS), and 0.5 M sodium chloride. Bacteria were first analyzed in tapping mode to determine their location and observe their morphology. Following this, the FV mode was used to create maps, and indentations at higher force loads were also performed using the point-and-shoot tool.

### Atomic force microscopy measurements

The surface analysis and mechanical characterization of living bacteria was performed with an AFM Multimode 8 from Bruker with a NanoScope V controller operated in fluid conditions throughout all experiments, using a pre-assembled fluid chamber with the appropriate solutions within a sealed O-ring. The instrument was operated in contact mode using MLCT probes from Bruker, cantilever D with a nominal spring constant of 0.03 N/m, and tip radius of 20–60 nm as provided by the manufacturer (Bruker), to study the morphological features of bacteria under different tested conditions. The deflection set point was adjusted during the measurement to optimize imaging conditions. Nanoindentations were performed using a maximum loading force of 500 pN for each nanoindentation applied in a pre-programmed grid of 48 points per line to quantify surface interactions and the mechanical response of bacteria. This allowed us to simultaneously acquire maps with enough resolution on the sample surface to identify individual bacteria and obtain the mechanical response from the membrane rigidity within a reversible regime. The advantage of mapping bacteria at low loading forces is that surface interactions between the tip and cell membrane can be analyzed, and information related to its adhesion or surface charge could be revealed with minimum damage to the mechanical integrity of the cell membrane. Before each experiment in FV mode, the cantilever was calibrated using the thermal-noise method which is integrated in the multimode AFM software and is briefly described next. First, the optical-lever sensitivity (OLS) of the cantilever was extracted. A force–distance curve was taken on a clean (previously cleaved) mica surface and a linear fit to the approach trace within the region following contact was performed to obtain the OLS from its slope. Afterwards, the cantilever was withdrawn above the surface and a thermal tune was performed to obtain the spectrum of the cantilever. A Lorentzian fit was then performed to the obtained peak and finally the cantilever constant was calculated.

**Figure 2:**
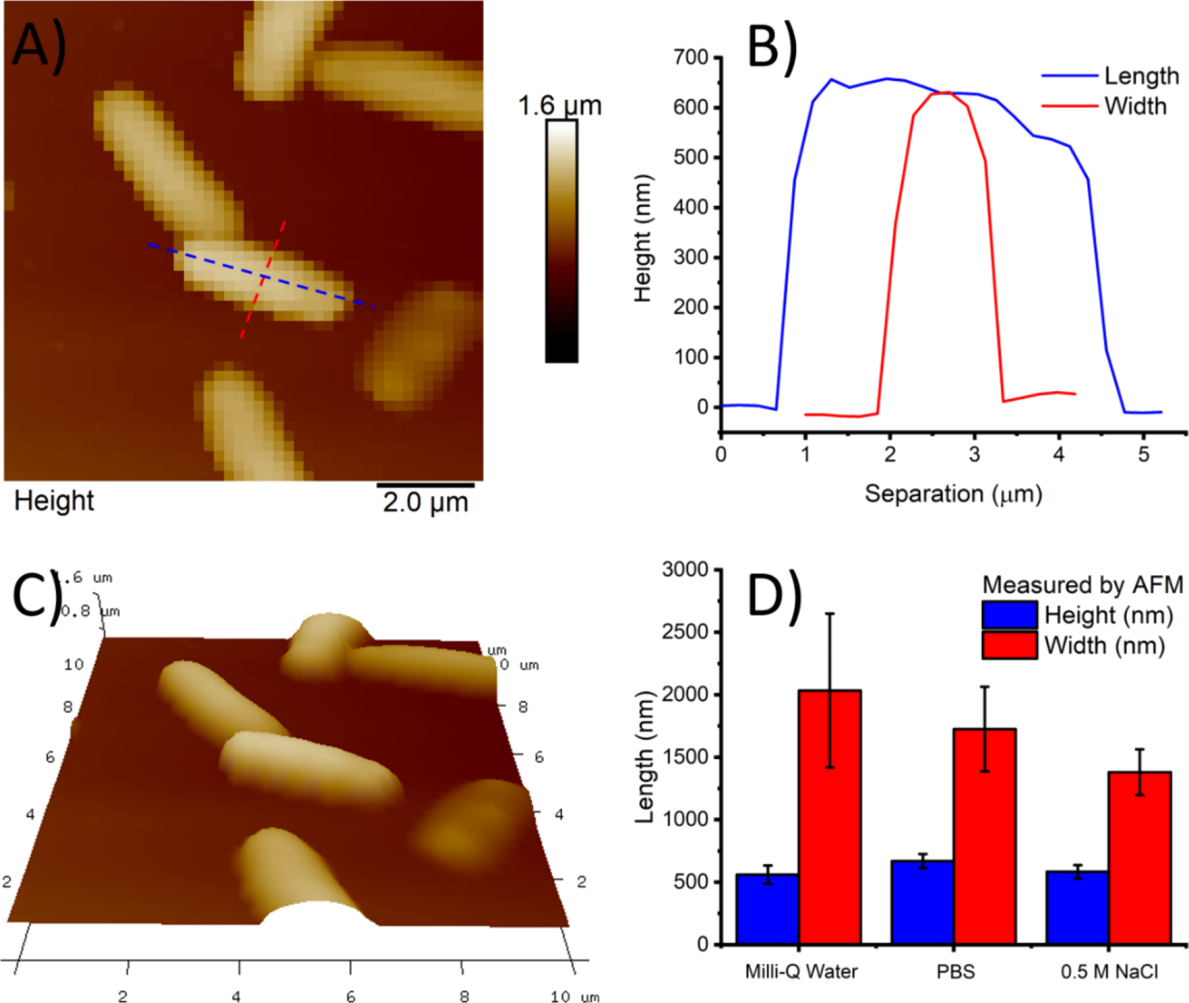
Mapping living bacteria with force–volume AFM. A) 2D height image of *Pseudomonas aeruginosa* obtained in FV mode where pixels indicating the location of single nanoindentations can be easily discerned. B) Cross-section profiles taken from both main axes of the bacteria shown in A) with dashed lines for length (blue) and width (red). C) 3D reconstruction of the image shown in A) shows how FV can also accurately represent the topography of the sample. D) Dimensions of PA obtained from AFM in different measured osmolarity conditions.

To take into account any potential change in the dimensions of PA at different osmolarities, the cross section tool from the NanoScope software was used to measure the long (length) and short (width) axes of the bacteria after being tested in FV mode. At least 20 bacteria were measured for each investigated condition. In AFM, the half-width distance is usually reported to take into account the influence of the AFM tip due its curvature close to the apex. A single measurement was taken along the length of each bacteria, while three measurements were performed and averaged to take its width. This is presented below in Figure 2A and Figure 2B.

All obtained images, FV maps, and force–distance curves were analyzed with the NanoScope analysis software 1.7 from Bruker. Once data for nanomechanical mapping at low loading forces was extracted, it was also attempted to produce maps at high loading forces of about 3 nN, as described below in the results section.

## Results

Before any nanoindentation measurements were performed, bacteria were first localized on the surface of poly-ʟ-lysine-coated mica. Supporting Information File 1, Figure S1 shows some 3D images of PA taken in contact mode when in PBS. Bacteria and the substrate can be observed. For a straightforward interpretation using quantitative nanomechanics in FV mode, the object to be mechanically tested must be clearly distinguished from a rigid substrate used as background. In this case, the thin layer of PLL (1–2 nm) on mica is expected to provide such background. Regions especially crowded with bacteria were intentionally avoided when applying FV. The capacity of AFM as a quantitative analysis tool when operated in FV mode is illustrated in Figure 1. Once a region has been selected with conventional AFM imaging, a grid is defined with a special resolution of points per line and tip velocity upon approach/retraction (see Figure 1A). Each approach/retraction cycle to a maximum load generates a force–separation curve analyzed in real time by the software. A color-coded map is obtained according to the physical parameters extracted from the curve, as shown in Figure 1B,C.

Figure 2 shows an FV image of the topography or height channel for several individual PA bacteria on the substrate. An estimation of the size of PA by AFM was made using the cross-section tool along both axes to obtain its length, width, and height, as shown in Figure 2A and 2B. With a resolution of 48 points or pixels per line, FV can also provide accurate morphological measurements for bacteria, as shown in the 3D reconstruction in Figure 2C (see also Supporting Information File 1, Figure S2). These measurements were taken for bacteria in the three investigated conditions (i.e., Milli-Q water, PBS, and 0.5 M NaCl) and the results are shown in Figure 2D. Larger values for the length and width of the bacteria are expected in AFM measurements when compared to other experimental techniques such as optical microscopy or electron microscopy. This is due to the fact that the finite size of the tip apex introduces an overestimation in the lateral dimensions of the imaged object but not of its height, where AFM excels in accuracy. From the plot in Figure 2D, we can observe that while its height remains around the same value of 500–700 nm, the change of its width shows a slight trend towards shrinkage of PA from hypotonic to hypertonic conditions. This might be a consequence or a first sign of readjustment of the cell volume when the adhered bacteria are abruptly exposed to different osmotic pressures.

**Figure 3:**
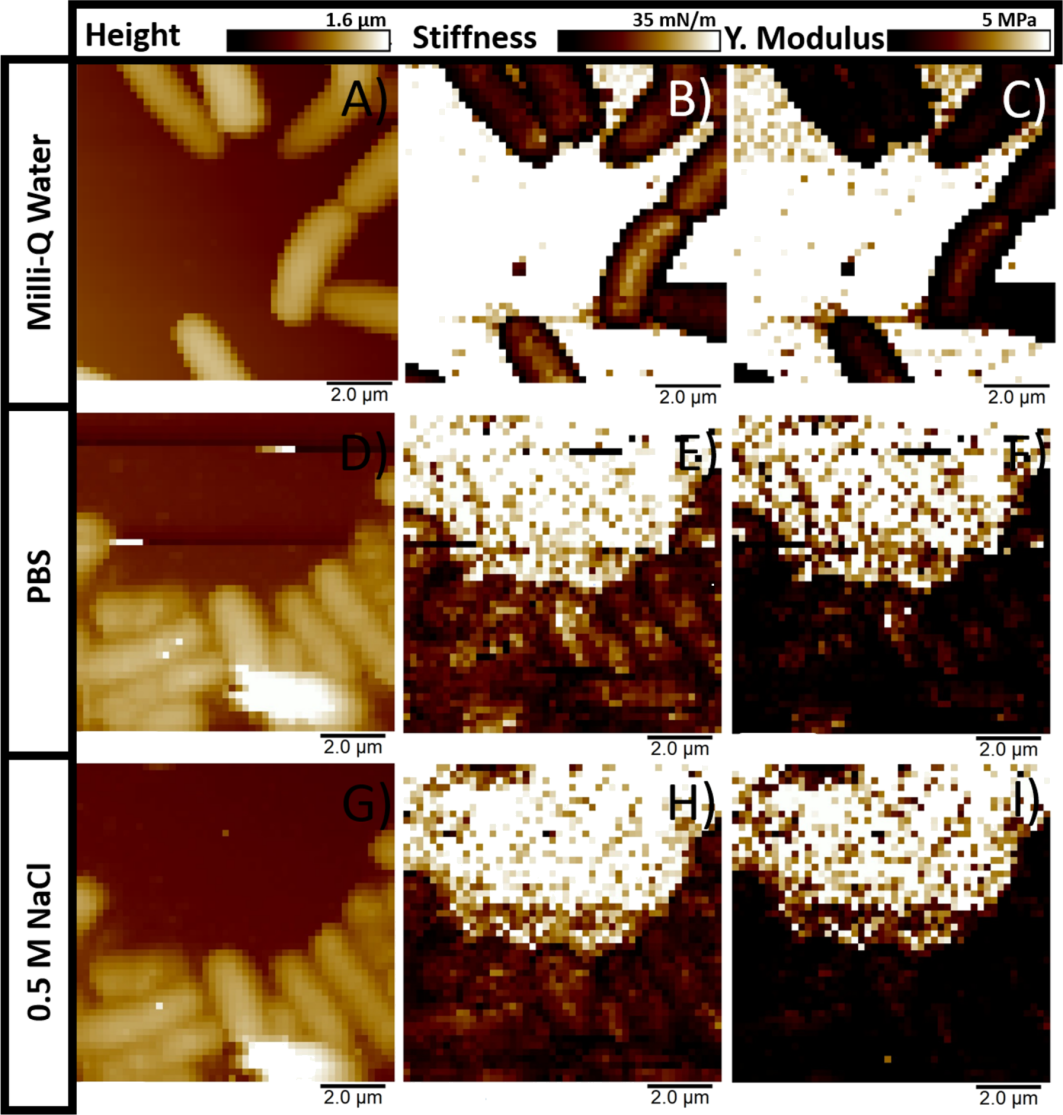
Nanomechanical maps of PA were obtained in FV mode for the three investigated conditions with a maximal loading force of 500 pN. The physical parameters shown are height (A, D, G), stiffness (B, E, H), and Young’s modulus (C, F, I). As observed, PA is identified on the surface in contrast with the background due to its different interaction with the AFM tip under the same loading force. Measuring conditions are given as rows, while colored scales for each extracted parameter are shown as columns.

In Figure 3, maps for three of the main physical parameters, height (*h*), stiffness (*k*), and Young’s modulus (*Y*) obtained in FV, are shown as columns, while the measuring conditions are given as rows. For the height channel in the first column, the identification of individual bacterium is possible even when they are aggregated. As expected, PA appears brighter than the substrate plane since the offset of the color-coded scale has been set for the best contrast and clarity for both. Bacteria appear dark to a brighter background for stiffness and Young’s modulus. In these cases, the offset in the color-coded scales has been set to 0–35 mN/m and 0–5 MPa, respectively, for an easier and more direct interpretation of the obtained values. The stiffness is obtained from the slope of the curve during compression by the AFM tip from the point of contact (*C*_p_) until it reaches its maximum predefined loading force. For this reason, the rigidity of the underlying substrate can be assumed to behave as an impenetrable substrate for the range of the applied loads. In the present case, the rigidity of muscovite mica represents a good approximation for the last assumption. However, the thin polymer layer of PLL applied to enhance the adhesion of PA is expected to introduce a certain but rather small degree of mechanical resistance. Visually, from the maps in the column for stiffness, it is difficult to discern a difference in the brightness on the surface of PA from the hypotonic (Milli-Q water) to isotonic (PBS) and finally to the hypertonic solution (0.5 M NaCl). Nonetheless, in the case of Young’s modulus, bacteria in the maps seem to become slightly darker with the increase in ionic strength.

Contrary to stiffness, the map for Young’s modulus is extracted after a mechanics model has been chosen in the analysis software and the appropriate parameters between the AFM tip and sample have been given. Although the apex of the tip used in this case is not a perfect cone, and the radius of the tip is about 10% of that of PA, analysis of the force response obtained in the force curves suggests that the Sneddon model provides a better description for the induced deformation than that of the Hertz model for the case when a spherical tip indents on an elastic planar half-space. Under the frame of the Sneddon model, a rigid conical indenter pushes a planar elastic semi-space characterized by a particular elastic modulus *Y*. The Sneddon model then provides a relation between the applied force load (*F*), the induced deformation (δ), and the modulus of elasticity (*Y*) of the tested material in the following way:


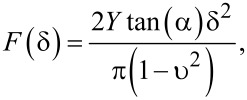


with α as the half angle distended by the apex of the AFM tip, and υ is a unitless factor known as the Poisson ratio, which takes values between 0 and 0.5. In the present study, we use υ = 0.49, as usually reported for biological samples [48–49].

**Figure 4:**
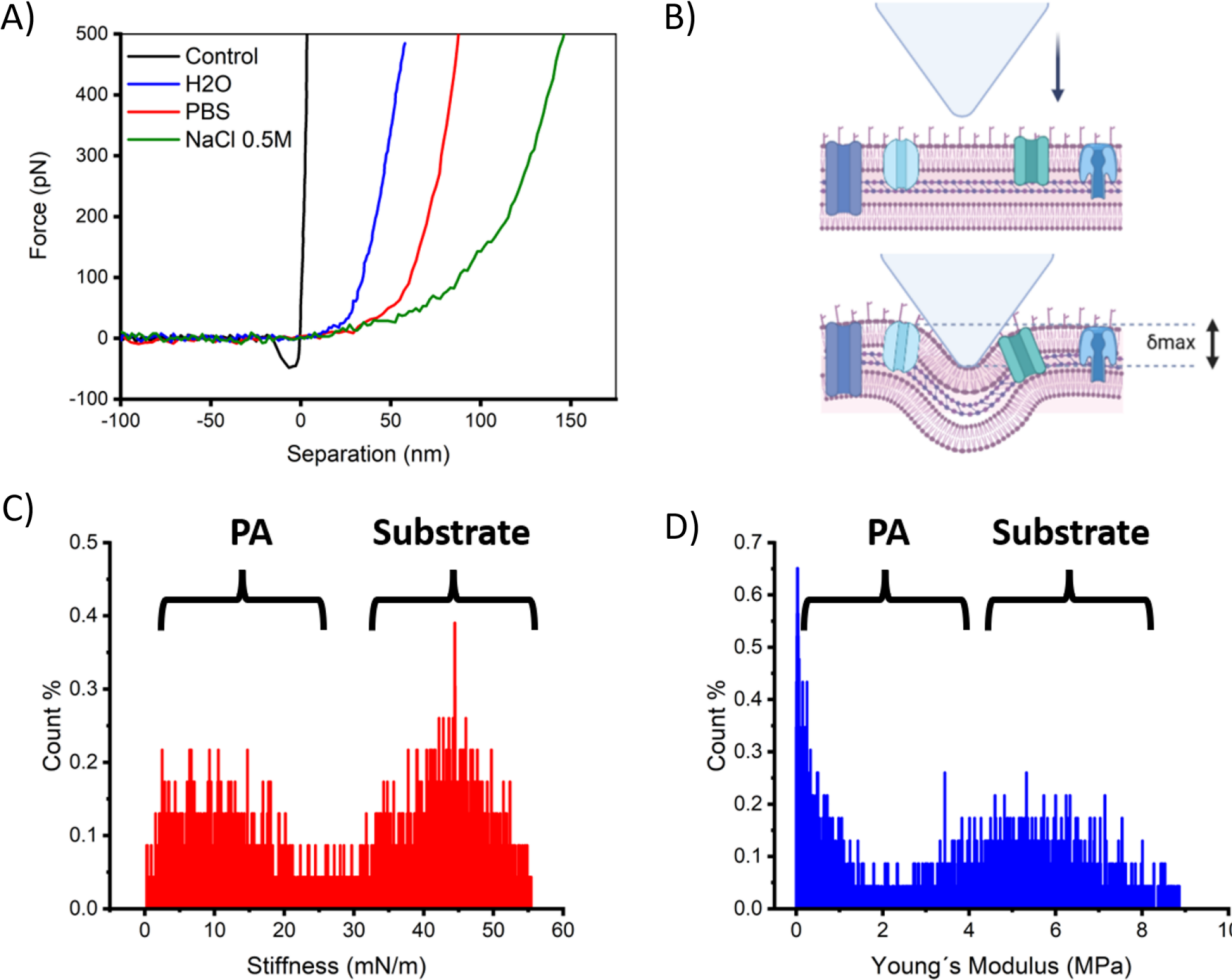
Degree of compression by nanoindentations in FV mode at low loading forces of 500 pN. In A) representative force–separation curves were obtained from the force response of PA indented under different tested conditions. Force curves were taken from the middle region of the bacteria to minimize variations arising from the finite size of the AFM tip interacting at the edges of the bacterial body. A control curve next to the bacteria is also shown as a reference for deformation (black line). In B), a drawing illustrates the outer membrane compression by the AFM tip (Created in BioRender. García-torres, L. (2025) https://BioRender.com/qynn0nv. This content is not subject to CC BY 4.0.). In C) and D), histograms for the stiffness and Young’s modulus values obtained from a map taken in FV mode in Milli-Q are shown, respectively.

Figure 4A shows the approach component of representative force–separation curves obtained on PLL and PA for each investigated condition for a maximal loading force of 500 pN. These curves show the mechanical response from the polymer layer on the mica surface and the outer membrane of PA. From the black line, it is easy to observe how the tip, upon contact, almost immediately attains full compression on the PLL layer, and no further deformation can be observed as the slope of the curve almost reaches verticality. The necessary deformation depth required to fully compress the PLL layer was 1–3 nm. An obvious difference in the force response is obtained on PA (blue, red, and green lines). For these low regimes of loading forces, it can be observed that the force response is nonlinear within the first 70 pN for Milli-Q water and about 100 pN for PBS. For 0.5 M NaCl, the curve remains nonlinear up to about 250 pN. For the maximal applied loading force of 500 pN, a maximal attained membrane deformation δ_max_ occurred at 55, 80, and 145 nm for Milli-Q water, PBS, and 0.5 M NaCl, respectively. Consequently, we observed a clear increase in the maximal induced deformation with an increase in the osmolarity. For this extent of induced deformation, the outer envelope of PA showed a reversible behavior in the mechanical response. Figure 4B shows a schematic representation of the AFM tip indenting on the outer membrane of PA and its maximum deformation δ_max_. A fit to the obtained force response using the Sneddon model in the NanoScope software for the curves shown in A) is given in Supporting Information File 1, Figure S3. These representative force curves have been selected from nanoindentations captured along the middle region of the longer axis of the bacteria in FV maps. This is of central importance since it was observed that the force response drastically changes according to the direction of the scanning in relation to the orientation of the bacteria. Especially at the edges, the influence of the finite 3D size and angle of the AFM tip became evident because indentation can take place perpendicularly, along the longer axis of PA, or in an intermediate diagonal direction of the bacterial body, which ultimately significantly alters the profile of the obtained force response. In Figure 4C,D, histograms from FV maps for the obtained values for *k* and *Y* are given for the case of Milli-Q water. The rest of the data is given in Supporting Information File 1, Figure S4. Although expected, a main feature in the obtained histograms for *k* and *Y* for all measured samples was the presence of bimodal distributions, which reflects the mechanical response from a homogeneous substrate coated at some percentage with bacteria. Therefore, one distribution corresponded to the mechanical response from the PLL-coated mica, while the other to PA. In the case of stiffness, both distributions appear Gaussian, with the substrate stiffness (*k*_s_) at higher values than that for the bacteria (*k*). Nevertheless, for Young’s modulus, only the distribution arising from the substrate (*Y*_s_) is Gaussian, while the one for the bacteria (*Y*) at lower values resembles a lognormal distribution. Supporting Information File 1, Figure S4 fits the obtained experimental data with Gaussian and lognormal distributions to obtain mean values. The obtained δ_max_ shows that in this low range of loading forces, the membrane rigidity is tested beyond the thickness of the bacterial outer cell wall, which, according to cryo-electron tomography, is about 25–30 nm [50]. In Table 1, the mean value obtained for the stiffness and Young’s modulus from the distributions are given and compared with mean values of *k*_PA_ and *Y*_PA_ intentionally selected from the middle regions of PA. The corresponding mean values for the maximum deformation or penetration δ_max_, attained by the AFM tip during compression on the central region of the bacteria, are also given. Table 1 shows that the mechanical resistance to deformation reflected in the stiffness remained almost unchanged when the mean values obtained from distributions for the different measuring conditions were compared. However, they differ from the mean values obtained from the middle region of bacteria. This accounts for the mechanical stability of the outer membrane of PA once a certain osmotic pressure is set. We can observe that mean values for *k* and *Y* obtained from the distributions were smaller than those obtained from the middle region of PA. This comparison also indicates how, in a nanoindentation map, the distribution overshadows the mean value taken where the geometrical conditions are usually preferred during mechanical testing since their number is just a small fraction of the entire set of indentations performed along a map. For mean values obtained along the central region of the bacteria, the stiffness also remained relatively unchanged for the different tested conditions. Still, we see a slight trend towards lower values from hypotonic to hypertonic solutions for the Young's modulus. This observation will be discussed below in the discussion section regarding the application of the mechanics model. Opposite to the Young’s modulus, the mean maximal induced deformation upon compression δ_max_ increases in the presence of a higher salt concentration.

**Table 1:** Mean values for stiffness and Young´s modulus obtained from distributions in FV maps and from the central region of PA.

Solutions	*k* (mN/m) from distributions	*Y* (MPa) from distributions	*k*_PA_ (mN/m) from the middle region of PA	*Y*_PA_ (MPa) from the middle region of PA	δ_max_ (nm) from the middle region of PA

Milli-Q	8.83 ± 0.46	0.51 ± 0.07	21.3 ± 4.9	1.13 ± 0.38	51.74 ± 10.94
PBS	9.32 ± 3.69	0.5 ± 0.08	17.6 ± 5.28	0.93 ± 0.39	88.5 ± 17.47
0.5 M NaCl	7.4 ± 0.17	0.14 ± 0.01	23.2 ± 5.36	0.69 ± 0.44	92.15 ± 27.85

Supporting Information File 1, Figures S5 and S6 show maps for the adhesion force between the AFM tip interacting with the substrate and bacteria. In Supporting Information File 1, Figure S5A–F a clear contrast in the adhesion force between the bacteria and the substrate can be observed for the case of Milli-Q water, where it was possible to quantify the adhesion force to the AFM tip as shown in the cross-sections in C) and F). When salt is introduced into the system, the contrast in the map is almost entirely lost, strongly suggesting that the electrostatic surface forces have been screened by the presence of ions, as shown in Figure S6 of Supporting Information File 1.

**Figure 5:**
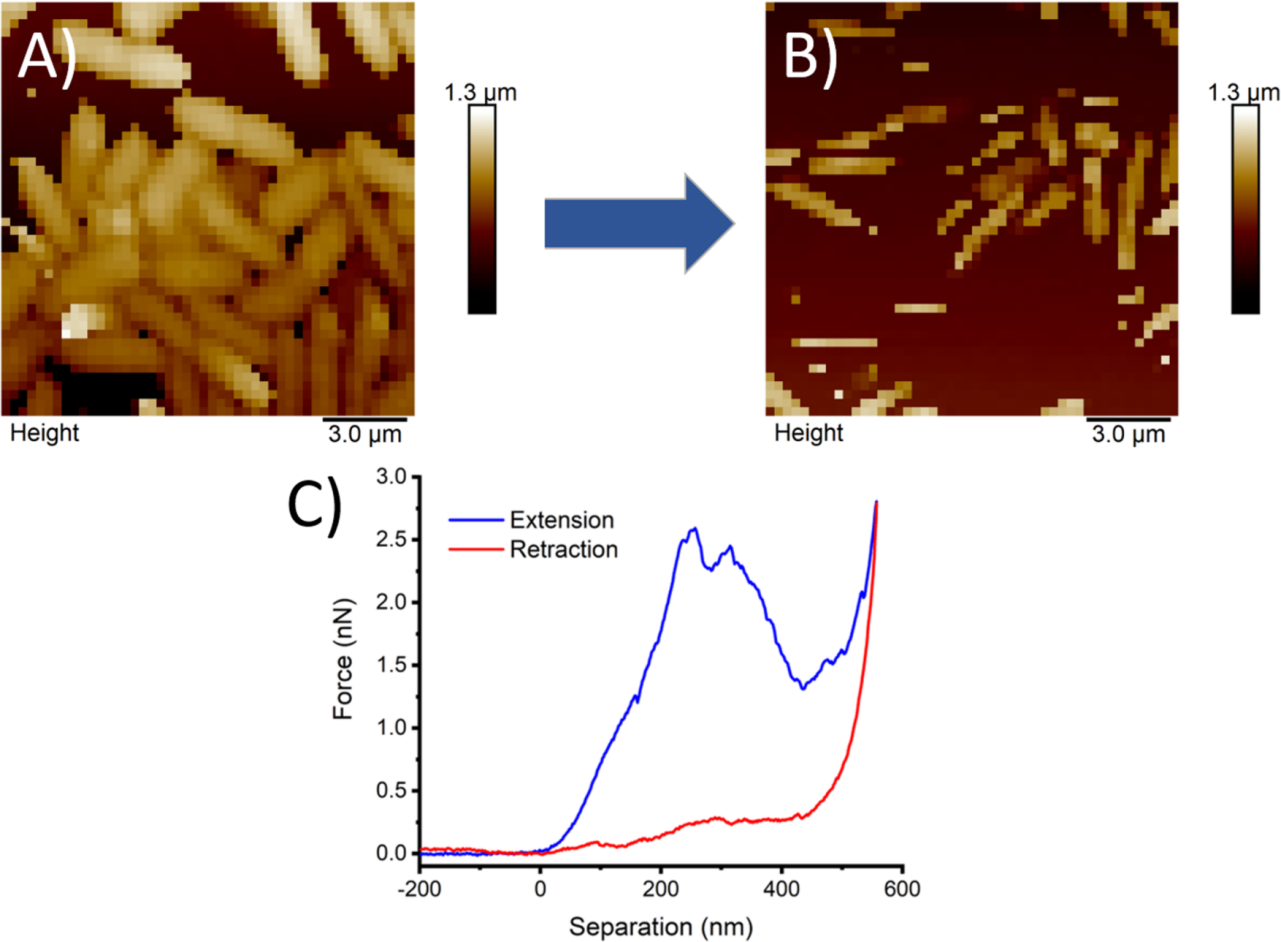
Force–volume maps were obtained at low A) and high B) loading forces in the same scanned region. The image clearly shows the entire destruction of previously mapped bacteria. C) A representative force–separation curve on PA where a drastic drop in the force is detected at about 2.5 nN.

Finally, in Figure 5A and 5B, it was demonstrated how applying repetitive compressions via nanoindentations at high loading forces in hypertonic solution led to the destruction of previously imaged bacteria. In Figure 5A, a region heavily coated with PA was mapped in 0.5 M NaCl with FV with 500 pN as the maximum loading force, and in Figure 5B, the same region was imaged under the same conditions but with a maximal loading force of 3000 pN. A complete loss of identifiable bacterial structures is obtained. These results demonstrate the incapacity of PA to maintain the structural integrity of the outer membrane when exposed to larger loads within the time scale of the present experiment, as shown in the force curve in Figure 5C. The maximal force that the membrane could withstand before rupture was 2.1 ± 0.5 nN.

## Discussion

Based on the FV mode of AFM, the results presented here test the mechanical resilience of bacteria as a whole under different conditions of external osmotic pressure with repetitive nanoindentations. It was observed that histograms for the obtained physical parameters from nanoindentation maps provide a full picture of the landscape being imaged. However, they can obscure relevant mean values when there is a geometry-dependent interaction between the AFM tip and the sample. From the many individual nanoindentations across the bacteria surfaces along each scanned line, only a few test the central region of interest, where mechanics models can better estimate the rigidity or elastic modulus. The different orientations of the rod-shaped morphology of PA on the substrate while being scanned by the quasi-conical AFM tip with a particular apex half-angle provide a broader spectrum of values on the nanoindentation map. Consequently, meaningful data is present but hidden within the statistical set. For this reason, mean values extracted from histograms were compared with data taken by point nanoindentations from the central region of the long axes of bacteria. Further analysis of these force curves is the focus of the following discussion. Upon compression by the AFM tip, PA showed two main regimes directly after contact. A first nonlinear regime following the contact point is commonly associated with the elastic response of the outer envelope. At the same time, for deeper deformations, a linear behavior in the force curve reflects the magnitude of internal turgor pressure *P*_t_ [51]. It is worth mentioning that for our case, only a handful of force curves, particularly in Milli-Q, presented a long tail upon approach, which frequently makes it difficult to identify the position of the contact point. This has been previously reported in other nanoindentation studies on bacteria, and its origin has been related to the action of repulsive electrostatic forces with long Debye screening lengths [44,52]. Our results obtained for the changes in the mechanical response of PA in the low deformation range under different external osmotic pressures are summarized in the parameters of stiffness (*k*), Young’s modulus (*Y*), and maximal induced deformation (δ_max_) (see Table 1). These results suggest that the mean values for stiffness remain almost unchanged for the tested conditions. However, mean values obtained for Young’s modulus show a shallow trend to decrease with higher osmolarities, particularly when point nanoindentations were used. On the other hand, a clear increase in the penetration depth δ_max_ is observed for higher osmolarities. This behavior of *k* and *Y* can be understood, considering the observed change in curvature in the force curves at the beginning of the indentation process and before the curve approaches linearity. The Sneddon model used here to extract the elasticity modulus is sensitive to these changes in curvature even when the slope within the linear regime at the final stage of the trace remains almost unchanged. The increase in δ_max_ (Figure 4a) from 55 to 145 nm accounts not only for this susceptibility of the outer envelope to deform at high osmolarities but also shows how the slope barely changes before it reaches the force threshold of 500 pN. These results are interpreted as follows. For the three investigated osmolarity levels, bacteria do not seem to be disrupted or damaged, at least from a morphological point of view when imaged in the low regime of loading forces or from its mechanical response to external deformation as given in the force curves. Consequently, these results suggest that in each tested solution, the tension exhibited by the outer envelope reflects the differences in the internal osmotic pressure built within the bacterial cell wall once the corresponding protein channels mitigate any threatening conditions to the integrity of the membrane (e.g., exceeding internal pressures due to drastic osmotic downshocks or shriveling caused by sudden shrinkage of the membrane in hypertonic solution). During exposure to a hypotonic solution, membrane lysis by osmotic shock is prevented by the rapid reaction of the MS channels, which act as safety valves which open as soon as the internal *P*_t_ causes an increase in the tension of the envelope above a specific threshold value. Then, it rapidly releases contained osmolytes to deflate the internal pressure and avoid membrane rupture. Using forward light scattering experiments, Çetiner et al. reported that the time required for PA to reach an equilibrium state after osmolyte release when exposed to drastic osmotic downshocks was about 150 ms [14]. In our experiments, PA was tested in different conditions after the solution was exchanged in situ in the liquid chamber of the AFM and allowed to settle. This time was about 5 min; therefore, is quite above the reaction time of PA to complete the osmolyte release. In our study, measurements were performed once these tension-triggered channels had already dissipated the exceeding internal pressure and reduced membrane tension below the activation threshold. On the other hand, a pronounced efflux of water leaves the bacterial body in hypertonic solutions. It drastically reduces the internal *P*_t_, resulting in severe dehydration of macromolecules inside the bacteria and the inner membrane envelopes. Lewenza et al. reported the treatment of PA with high salt concentrations and showed that PA develops plasmolysis bays due to shriveling in hypertonic solutions [53]. These bays are generated by a separation between the inner and outer membrane envelopes due to water depletion. As a protective countermeasure of PA against osmotic upshock to avoid collapse by shrinkage, it is known that cationic ions such as K^+^ are imported by special protein transporters and accumulated by bacteria to maintain homeostasis against hazardous external concentrations of sodium ions [54]. Also, other accumulated or internally synthesized osmoprotectants such as *N*-acetylglutaminylglutamine (NAGGN) and glutamate contribute to its survival under critical hypertonic solutions, while hydrophilins are also believed to confer protection [55]. Analysis of our force curves with maximal loading forces of 500 pN in hypotonic and hypertonic solutions yielded mean values for the maximum penetration depths (δ_max_) of about 52 and 92 nm, respectively. In contrast, for PBS, an intermediate value was obtained. Considering the above information regarding the states of swelling or plasmolysis, our results for the stiffening and softening of the outer envelope can be explained in terms of the internal pressure *P*_t_. Although membrane rigidity is an intrinsic property of the two-dimensional material, membrane tension is not. Instead, membrane tension depends on the forces acting on the membrane, which is directly influenced by the internal turgor pressure [56]. As depicted in the force curves for the case of hypotonic solution, the combination of a high curvature with shorter δ_max_ strongly indicates a higher membrane tension as a result of osmolyte release in the final equilibrium phase by the MS channels, which is consistent with values similar or slightly above to those obtained in PBS. Meanwhile, in the hypertonic solution, the curvature at the beginning of the curve is lower, and further deformation is required to attain the linear behavior. Based on our force spectroscopy results and reported fluorescence microscopy images showing plasmolysis bays in PA under hypertonic conditions, we speculate that this mechanical response is explained by the detachment between the outer and inner bacterial envelope caused by dehydration [53]. A loose outer envelope that has lost its tight connection with the inner envelope due to severe water depletion could provide a consistent explanation for the lower mechanical resistance or softening observed during the first steps of deformation. Consequently, additional deformation would be necessary before reaching the mechanical resistance transmitted from the inner envelope.

As stated above, the contribution to the steepness in the linear regime in the force curves reflects the *P*_t_. In our study, PA seems to keep its mechanical response within the same values in hypotonic and hypertonic solutions, which shows its mechanical fitness after the equilibration process to some extent. The elastic deformation of PA in the frame of AFM nanoindentation can be modeled as the simultaneous compression of two spring elements in series with different stiffness, where the system cantilever+tip with stiffness *k*_cant_ compresses PA with stiffness *k*_PA_. Thus, in a force curve, the slope in the linear region represents the effective action of both springs acting together, which can be expressed as *k*_eff_. Applying Hooke’s law for a system of two springs being compressed in series and solving for *k*_PA_, we obtain *k*_PA_ = (*K*_cant_ · *K*_eff_) · (*k*_cant_ − *k*_eff_)^−1^ [57]. Table 1 shows the mean values obtained from this calculation once the effective stiffness values were extracted from the force curves. Measurements and a detailed analysis of membrane deformation by AFM nanoindentations on the Gram-negative bacterium *Magnetospirillum gryphiswaldense* were first published by Arnoldi and Boulbitch [51,58]. Their study revealed a stiffness value for the membrane in the 0.04–0.07 N/m range. Their theoretical analysis allowed them to use this value to estimate turgor pressures within the 85–150 kPa range. To avoid the complex mathematical analysis of that first study on membrane elasticity, Yao and coworkers presented a tension-dominated model to derive the internal turgor pressure [52] of the Gram-positive and negative bacterium from the linear region of the slope obtained from force curves during deformation. For Gram-negative bacteria, they found that *P*_t_ increases an order of magnitude when bacteria are tested in distilled water (≈1.9 × 10^5^ Pa) compared with growth medium (0.1–0.12 × 10^5^ Pa). Under the framework of the tension-dominated model developed by Yao and coworkers for rod-shaped bacteria such as PA, we can estimate *P*_t_ in our present study. Considering a mean height for PA of about 600 nm, an inner radius of 250 nm, which considers the presence of the double membrane in Gram-negative bacteria, and a mean stiffness of *k*_B_ = 20 mN/m, we obtained a turgor pressure of *P*_t_ = 12.1 kPa. This obtained value for *P*_t_ is in the same order as that reported by Yao for the growth medium. The higher value they reported for the stiffness in distilled water directly impacted the estimation of *P*_t_, as all other parameters were practically unchanged. We do not observe this substantial difference in stiffness between PBS and Milli-Q water in our measurements. Aware that both studies were performed with a similar experimental setup using a fluid chamber in the AFM, we can argue that time scales between measurements and between solutions were similar. We cannot explain the steeper slope reported in distilled water in that study. Furthermore, according to Çetiner et al., a rapid osmolyte release takes place within the first 150 ms following dilution, which should not only decelerate swelling but also return membrane tension below a threshold value during cell equilibration [14]. On the other hand, our reported values for Young’s modulus seem to capture the combined features in the force curve, meaning the curved region and the linear response. Altogether, our study suggests that the induced nanoindentations reflect different states of tension of the outer envelope at shallow deformations for the different tested solutions. Still, a similar stiffness arising from the remaining contents dominates for larger deformations. We associate this membrane tension and mechanical response with the equilibration time of the bacteria once MS channels have dissipated the osmotic gradient.

Care must be taken when quantifying the strength of adhesion forces via repetitive nanoindentations with the AFM because the risk of tip contamination is always present, and also because the sensitive nature of the intermolecular forces during the measuring conditions (pH, ionic strength, temperature, etc.) can easily affect their magnitude. This was reflected in the obtained adhesion maps (see Figures S5 and S6 of Supporting Information File 1). In Milli-Q water, it is well known that a silicon nitride AFM tip is negatively charged. Consequently, upon close contact, a negatively charged AFM tip will experience on one side an attractive force towards a positively charged surface given by the cationic PLL and on the other side a repulsive force arising from the negatively charged bacterial membrane. In Milli-Q water, electrostatic double-layer forces strongly enhance the magnitude of adhesion forces at close range. This effect can be observed in the force curve shown as the control in Figure 4A taken in Milli-Q water on the substrate coated with PLL. The black line shows the presence of a jump-in, indicating the existence of a pulling force ≈15–20 nm before contact. The obvious contrast in the map of the adhesion channels together with the cross-sections shown in Supporting Information File 1, Figure S5 indicate the magnitude of *F*_A_. On PA, a negative *F*_A_ or repulsion of about 25 pN was obtained, while a positive adhesion *F*_A_ of about 50 pN on the PLL coat was measured. These results are consistent with the information gained by ξ-potential measurements of PA when measured in Milli-Q water, where a negative surface charge for PA of about −25 mV was obtained (data not shown). Introducing salts into the solution resulted in the electrostatic screening of the double-layer forces, which resulted in the cancelation of long-range forces upon approach. The implication of this is shown in the adhesion maps of Supporting Information File 1, Figure S6, where a loss in the strength of adhesion forces started to occur in PBS while in 0.5 M NaCl it was even more pronounced.

## Conclusion

We performed shallow (500 pN) nanoindentations to quantify the changes in rigidity of the outer cell wall of *Pseudomonas aeruginosa* in hypotonic, isotonic, and hypertonic conditions. Force–volume AFM demonstrated its capacity for testing the mechanical properties of multiple bacteria at once. This mechanical nanoscale mapping allowed us to successfully discriminate the minute variations in the surface topography of bacteria (*h*), their mechanical resistance to external deformation (*k*), an estimation of their elasticity modulus (*Y*), and their adhesive behavior towards the AFM tip to the substrate (*F*_A_). Generated maps from nanoindentations indicated that PA tolerates drastic changes in osmotic pressure from isotonic (0.35 Osm) to hypotonic (<0.05 Osm) or hypertonic (1 Osm) solutions without showing serious structural or morphological damage of the bacterial bodies. Deformation of PA by a pseudo-conical tip resulted in force curves with two main features, mainly a first nonlinear region we associated with the magnitude of membrane tension followed by a linear region related to the internal turgor pressure. Our results showed that for the time scale of our experiments, the stiffness given by the linear regime of the curves did not significantly change the range of tested osmolarities. Application of previously developed models for AFM nanoindentation that relate the stiffness of a rod-shaped bacteria under deformation to its internal turgor pressure was used. The obtained values were consistent with those reported for Gram-negative bacteria in growth medium but inconsistent with those reported in hypotonic solutions. We argue that osmotic shock and potential cell lysis are prevented by the rapid reaction of MS channels to low osmolarities, which also decreases membrane tension. Decompression of exceeding internal pressures within PA is then reduced, and obtained values for *k*_PA_ and *P*_t_ represent the equilibration phase of PA which counteracts exceeding swelling. This demonstrates the remarkable dynamic capacity of the MS channels to rapidly mitigate hazardous internal pressures in highly diluted solutions. Therefore, we expect high membrane tension values to occur but in a very narrow period (within ≈100 ms after exposure), making it challenging to monitor AFM experiments. However, it was observed that the nonlinear region was affected by changes in osmolarity, providing a soft mechanical response for hypertonic solutions. We conclude that this initial weak mechanical response to deformation originates from a loose outer membrane due to dehydration, known as plasmolysis bays. Despite this, PA still provided a considerable mechanical response consistent with an internal turgor pressure for larger deformations. These investigations using nanomechanical mapping with FV AFM unravel fine mechanical parameters of very resilient bacteria with high adaptability to diverse environments. They also highlight the potential of the experimental approach to study the activity of rationally designed molecular channel inhibitors that can specifically bind channel proteins.

## Supporting Information

File 1 Additional figures.

## Data Availability

Data generated and analyzed during this study is available from the corresponding author upon reasonable request.
