# Supplementary material for: Mechanical stability of individual bacterial cells under different osmotic pressure conditions: a nanoindentation study of Pseudomonas aeruginosa
Source: Beilstein J Nanotechnol. 2025 Jul 21;16:1171–83. doi: 10.3762/bjnano.16.86 (PMC12302410; doi:10.3762/bjnano.16.86)
Supplement: File 1 — Additional figures. [file Beilstein_J_Nanotechnol-16-1171-s001.pdf]

BEILSTEIN JOURNAL OF NANOTECHNOLOGY

Supporting Information

for

Mechanical stability of individual bacterial cells under different osmotic pressure conditions: a nanoindentation study of *Pseudomonas aeruginosa*

Lizeth García-Torres, Idania De Alba Montero, Eleazar Samuel Kolosovas-Machuca, Facundo Ruiz, Sumati Bhatia, Jose Luis Cuellar Camacho and Jaime Ruiz-García

Beilstein J. Nanotechnol. **2025,** *16,* 1171–1183. doi:10.3762/bjnano.16.86

Additional figures

License and Terms: This is a supporting information file under the terms of the Creative Commons Attribution License (https://creativecommons.org/licenses/by/4.0). Please note that the reuse, redistribution and reproduction in particular requires that the author(s) and source are credited and that individual graphics may be subject to special legal provisions.
The license is subject to the *Beilstein Journal of Nanotechnology* terms and conditions: (https://www.beilstein-journals.org/bjnano/terms)

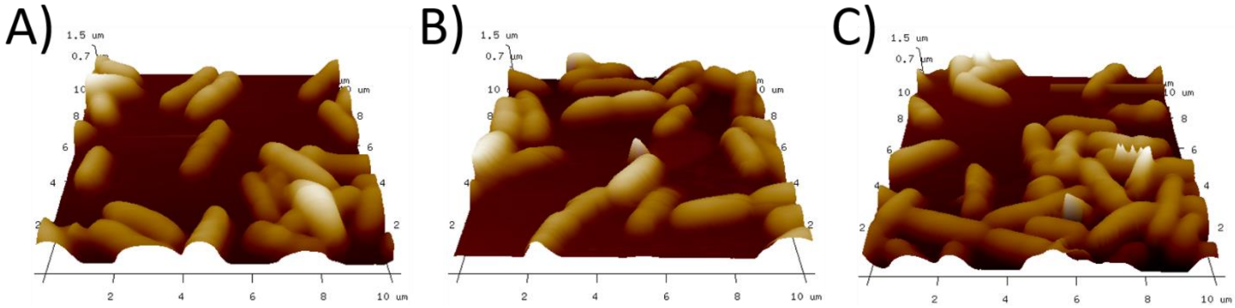

Figure 1: 3D AFM reconstruction images of PA on substrate taken in contact mode in PBS.

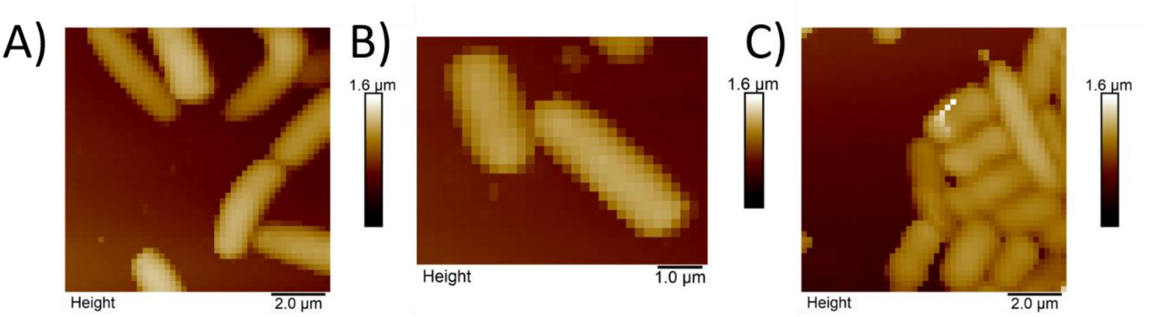

Figure 2: Height channel shows 2D maps of PA as obtained in force−volume mode. A) in Milli-Q water, B) in PBS, and C) in 0.5 M NaCl.

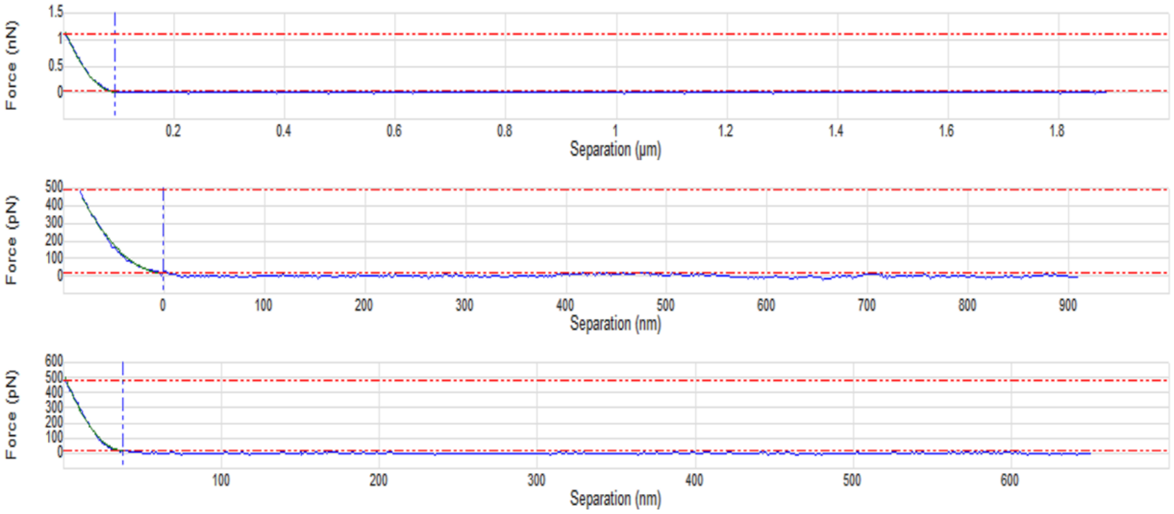

Figure 3: Experimental curves taken from the central region of PA and fitted with the Sneddon model using the software NanoScope 1.7. The blue continuous curve is the extension trace during nanoindentation, the green overlapped curve is the fitted function from the Sneddon model. The discontinuous vertical blue line indicates the point of contact from which the fit is taken, and the horizontal segmented dashed lines are the lower and upper limits of the fit.

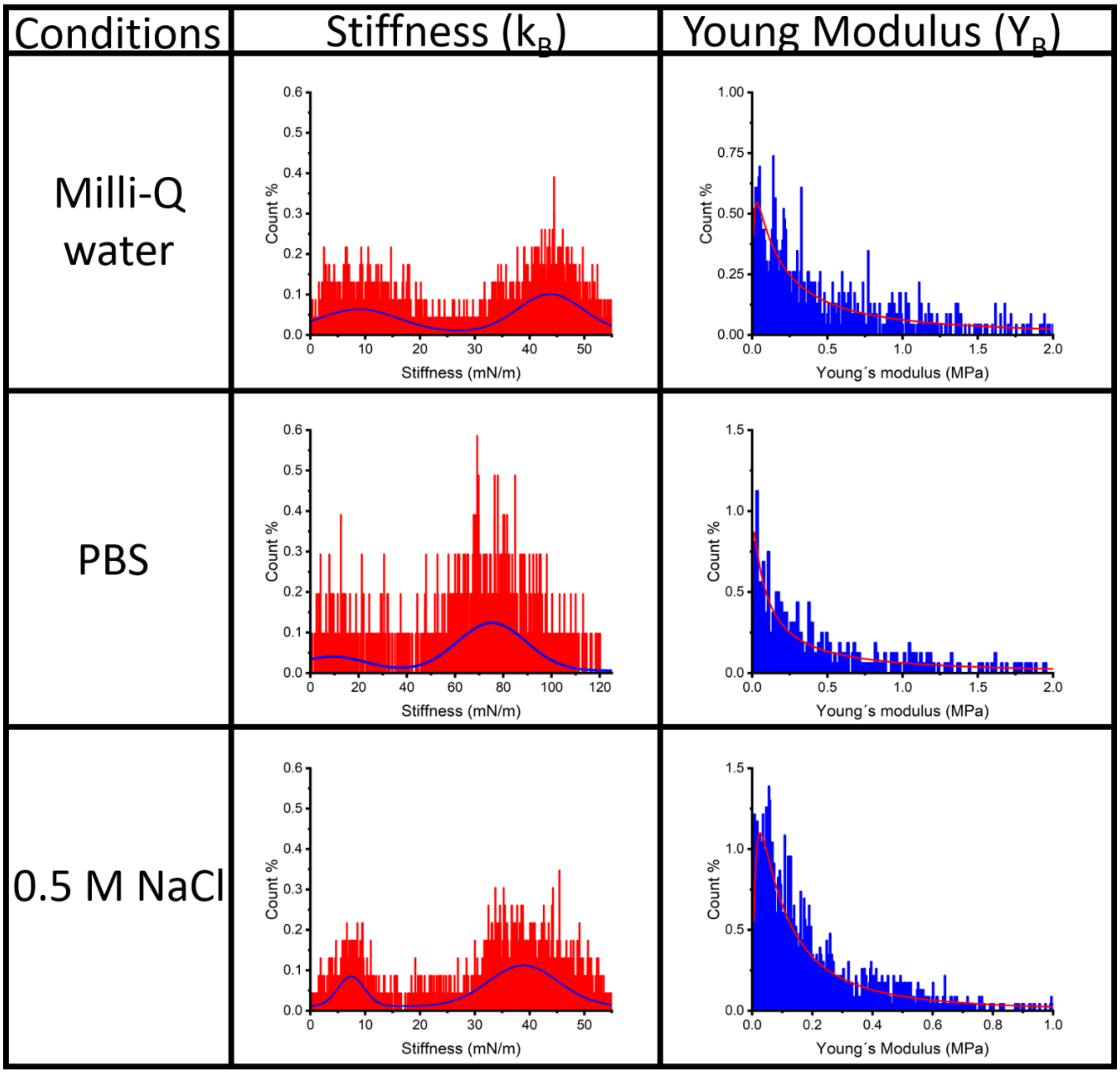

Figure 4: Histograms for the stiffness and Young's modulus obtained in force–volume mode from nanomechanical mapping for the three different investigated conditions. Distributions for stiffness (red distributions) have been fitted with Gaussians (blue lines) while for the Young's modulus (blue distributions) the fitting was done with log-normal functions (red lines).

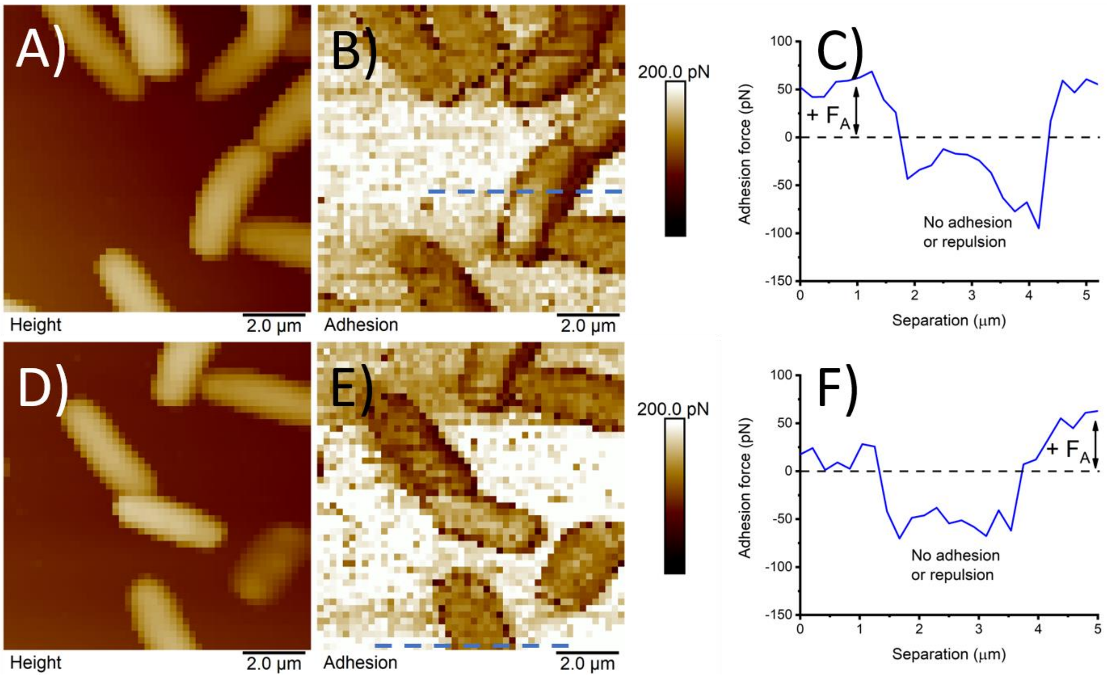

Figure 5: Height and adhesion channels of PA taken in force volume in Milli-Q water. For the sake of comparison, height channels have been added. A) and D) 2D force volume maps of height, B) and E) force volume maps of adhesion, and C) and F) cross-section profiles from the adhesion maps in B) and E) that show the difference in adhesion from the tip to the substrate and to the bacterial surface.

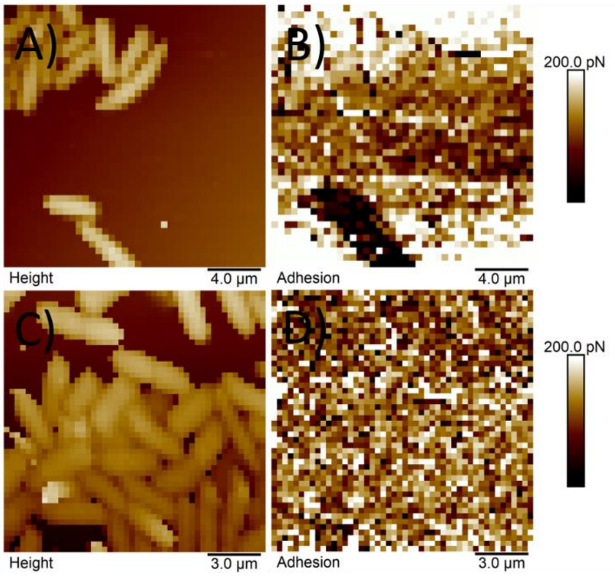

Figure 6: Height and adhesion channels of PA taken in force–volume mode. A–B) in PBS and C–D) in 0.5 M NaCl. For the sake of comparison, height channels have been added.
